# Catalyzing momentum in male contraceptive development^†^

**DOI:** 10.1093/biolre/ioab208

**Published:** 2021-11-05

**Authors:** Logan M Nickels, Kevin Shane, Heather L Vahdat

**Keywords:** Contraception, reproductive biology, grants, fellowships, research support, male contraception, spermatogenesis, sperm motility, sperm function

## Abstract

Globally, nearly half of all pregnancies are unplanned. Male contraceptives offer the potential to decrease unintended pregnancy and introduce contraceptive equity, but decades of research have yet to bring a novel product to market. New funding avenues from the philanthropic sector seek to stimulate research in male contraceptives through investments, grants, and support for trainees alongside other programs that encourage product development and ultimately commercialization. This Forum outlines the purpose of and funding opportunities provided by Male Contraceptive Initiative, a funding agency and non-profit focused on the advancement of non-hormonal, reversible contraceptive technologies for those who produce sperm.

## Teaser

Male contraception offers unrealized potential for improving global health and well-being, and increased research support is available to the reproductive biology community that facilitates further study in this important realm.

## Graphical Abstract

**Graphical Abstract:**
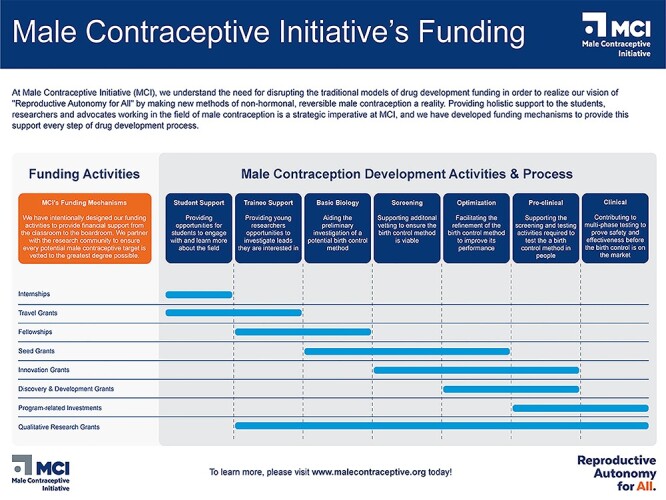

## Forum

Globally, nearly half of all pregnancies are unplanned. Unintended pregnancies lead to a cascading wave of undesirable outcomes such as decreased educational attainment and economic instability among women, their male partners, and their offspring [1]. The advancement of contraceptive technologies has mitigated these outcomes to some degree, particularly in high-income countries, but unmet need persists across geographies and social strata [2].

To-date, contraceptive development has largely focused on new options for cisgendered women, or those with uteruses, but cisgendered men, or those who produce sperm, have not seen significant advancement in contraception since the vasectomy, a relic of the early 20th century. The second-most recent innovation in male contraception was the condom several centuries before that. This lack of advancement is even more striking in that vasectomy is intended to be a permanent method, and the condom is associated with a real-world failure rate of approximately 15% [3]. The lack of development of novel male methods is in spite of global data indicating a significant demand for new forms of contraception and subsequently a very large market for male methods [4].

Novel methods of contraception for sperm-producers would not only provide a means of addressing the inequity in contraception, but have the potential to reduce unplanned pregnancies, foster greater relationships, provide a means of engaging men in a provider setting, and help to address the gender gap in healthcare [5, 6]. Contraception can also reasonably be applied to most, if not all of the UN Sustainable Development Goals [7].

In order to address this pressing global issue, Male Contraceptive Initiative (MCI) was established as 501(c)3 charitable organization to advance the development of contraceptives for those that produce sperm through advocacy and direct research funding. MCI provides support to the global research community in the academic, public, and private sectors working on non-hormonal, reversible methods of male contraception (NHRMC). MCI works towards the vision of “Reproductive Autonomy for All” through a variety of programs including direct research grants to primary investigators, program-related investments, and support for trainee investigators.

Despite the importance of NHRMC, many stakeholders have focused reproductive research on pressing issues of infertility and disease rather than contraception. To-date, MCI has awarded over $6 million towards research and development across over 50 awards and has designed programs to stimulate interest in NHRMC among the research community as well as address research projects across the development spectrum.

Within fields of andrology and reproductive biology, MCI intends to promote contraceptive research by providing support for NHRMC projects that span the range from basic science through clinical studies. MCI support is ultimately intended to facilitate the development of a spectrum of products with a range of characteristics that will meet the needs of a variety of future users. Projects currently supported by MCI include potential drugs and devices with long-acting, daily, and on-demand characteristics as well as a diverse slate of potential user delivery options. MCI makes open calls for proposals annually, and has funded multiple grants each year since 2017. More information can be found on the website www.malecontraceptive.org.

Generally speaking, research projects funded by MCI focus on a validated contraceptive target and have a viable pathway towards commercialization. However, MCI is also uniquely positioned to shift and scale funding for early-stage projects as well as mature projects. Multiple projects supported by MCI have demonstrated progress through the pipeline and advanced into preclinical or clinical phases of development.

Crucial to the development of NHRMC, as for any scientific field, is support for early-stage research as well as young researchers. Such support facilitates growth and sustainability as trainees advance to establish their own research programs. Fellowships and other support for trainee investigators can facilitate not only a pathway towards scientific progress, but also incentivize long-term growth in a traditionally small field.

At MCI, trainee support programs include a fellowship program, where graduate or postdoctoral trainees write and carry out a research proposal; internship programs for graduate and undergraduate public health students; and a program that facilitates travel, professional development, or other career advancement opportunities for young investigators who focus on NHRMC. Fellowship and internship programs occur on an annual cycle with application deadlines in the spring. Fellowships generally offer 2 years of support with stipend levels comparable to NIH institutional training grants, and other programs that offer career advancement opportunities are open on a rolling basis. All prospective applicants are encouraged to visit the MCI website for more information or contact MCI directly for further details.

Recipients of MCI’s trainee support have included SSR members and have had a variety of positive outcomes including awards, presentations, publications, and career opportunities in male contraception.

One point of MCI’s research strategy is to facilitate the de-risking of novel contraceptive projects that may require a certain fidelity of data or proof-of-concept for follow-on funding. By investing in scientifically sound work on promising targets, devices, or compounds, multiple MCI projects have been able to address key questions and attract follow-on funding from other sources, public and private.

Recipients of MCI support can also be availed to project-specific expertise and a network of potential collaborators or downstream developers, depending on the needs of the project. In addition to specifically supporting research and development, MCI has provided support for qualitative research and market research which assess the perceptions and preferences of NHRMC among men and their partners. MCI also supports the development of tools and techniques that can be applied broadly to the field of reproductive biology to advance NHRMC research.

An increased level of focus from the reproductive biology community on NHRMC, as well as contraception as a whole, is required to drive innovation in a space that has badly trailed other advances in healthcare. Agencies, philanthropic organizations, and other entities have the opportunity—and arguably the moral imperative—to incentivize growth and development, especially given the many recent advances that have begun to spur development across the male contraceptive pipeline [8].

Male contraception has the ability to have a major impact on the staggering unintended pregnancy rate and the cascade of associated negative outcomes. In addition, male contraception can increase gender equity and equalize contraceptive responsibility. Novel forms of male contraception can create a means of engaging men in a provider setting and facilitate a pathway to address the well-known gender gap in healthcare. By continuing and deepening the investment in novel forms of contraception, and specifically novel methods for men, we can simultaneously address a public health crisis, work towards a more equitable future, and develop appealing products that meet the needs of a diverse range of users.
